# Melting Temperature and Change of Lamellar Thickness with Time for Bulk Polyethylene

**DOI:** 10.6028/jres.067A.046

**Published:** 1963-10-01

**Authors:** James J. Weeks

## Abstract

The melting temperature of linear polyethylene has been obtained as a function of the time and temperature of crystallization. Recrystallization was minimized by a special melting procedure. By interpreting the melting points as characteristic of a given lamellar thickness, it was found that the thickness of crystals of appreciable age increased linearly with the logarithm of their time of existence. The lowest melting (i.e., thinnest) lamellae in a given specimen may be assumed to have either existed for only a short period of time, or to have been impeded in their growth in the chain direction, and they were found to have an estimated thickness close to that predicted by recent kinetic theories of polymer crystal growth with chain folding.

## 1. Introduction

Linear polyethylene is known to crystallize from its melt into lamellar structures which range in thickness from less than one hundred to several hundred angstroms [1–5]^1^. The extreme thinness of these lamellar crystals causes their melting points to be depressed below the equilibrium melting temperature, $T_{m}^{0}$, by amounts which are easily measurable. Thus, one can use the observed range of melting temperatures to obtain information concerning the distribution of lamellar thicknesses in a crystallized specimen.

Theoretical studies [6, 7] have indicated that the lamellar thickness (“step height”) of a growing polymer crystal should initially be equal to $l_{g}^{*}$, the thickness of the critical-size nucleus for maximum growth rate. Since a crystal with this dimension would melt only slightly above the crystallization temperature, *T_x_*, and since polyethylene is known to melt approximately midway between *T_x_* and $T_{m}^{0}$ [8], one concludes that the crystals have thickened subsequent to their original formation from the melt [9]. The exact relationship between *l*, the step height of a mature lamella, and $l_{g}^{*}$ is of interest in connection with an extrapolation method for obtaining $T_{m}^{0}$ [8] as well as for a detailed understanding of polymer crystal growth as controlled by nucleation mechanisms.

In this paper, the melting temperatures of the lamellae, or portions thereof, in a given specimen are reported as a function of the time and temperature of crystallization. By assuming that the melting temperature of linear polyethylene depends primarily on the lamellar thickness, one may correlate an observed melting point with the thickness of a lamella which was formed at a known temperature. However, in doing this, certain precautions must be taken to minimize changes in the crystal geometry prior to actual melting. Some of the factors that may affect the observed melting range of a sample are mentioned in the next section, and the extent to which these factors influence the choice of a melting procedure is indicated.

## 2. Factors Affecting the Observed Melting Temperature

### 2.1. Recrystallization

The melting of unstable crystalline regions of small size followed by the crystallization of the newly formed melt on existing stable nuclei is termed *recrystallization* in this paper. The occurrence of recrystallization makes it very difficult to obtain the complete distribution of melting points which characterize the crystals present in a given sample [10]. Polyethylene samples that are crystallized by quenching or slow cooling from the melt to room temperature will have crystallized to a considerable extent at high degrees of undercooling from $T_{m}^{0}$. Since crystallization at low growth temperatures produces very thin lamellae, such samples will contain a large fraction of low-melting crystals. These low-melting crystals will produce much crystallizable liquid and myriad nucleation sites if the specimens are warmed slowly through the melting range. Simultaneous melting and recrystallization at high growth temperatures will bias the observed melting curve toward the higher melting temperatures [8, 11]. As pointed out by Chiang and Flory [12], recrystallization in polyethylene is minimized by crystallizing at elevated temperatures.

The need to avoid recrystallization, or continued isothermal crystallization after a fixed time, has led to the adoption of a melting procedure wherein the sample is transferred from *T_x_* directly into a bath at a higher temperature where the spherulitic growth rate is negligibly slow. In selecting this temperature, one should bear in mind that recrystallization can more rapidly produce crystalline material than can ordinary isothermal crystallization from the melt at the same growth temperature. This has been clearly demonstrated by Gubler, Rabesiaka, and Kovacs [13] and probably is a result of the larger number of nuclei (unmelted regions of the lamellae) that are present in a sample undergoing recrystallization.

### 2.2. Isothermal Thickening of the Lamellae

The increase of the lamellar thickness of polyethylene crystals that may occur on storage at constant temperature is called *isothermal thickening.* It is presumed to take place without previous melting of the crystallites and, to a first approximation, not to increase the volume of the crystal but only its thickness. A similar slow-thickening process, wherein the lamellae are not melted, can of course take place while a specimen is being warmed.

Since the crystals of polyethylene do not possess a minimum surface free energy per unit volume when they are first formed during a crystallization process, there is a thermodynamic driving force which tends to increase the thickness of the crystals and thereby minimize the total free energy. The minimum total free energy of a single polyethylene crystal of specified volume could be achieved only if the lateral area of the crystal were about eight times as great as the chain-fold surface area (the ratio of the surface energies is about four to one [9]). No macroscopic single crystals have been observed having a shape approaching the equilibrium shape; thus, one may assume that the tendency for increasing the step height exists at all temperatures below the melting point, including *T_x_.*

As a result of an increase in thickness, the melting point, *T_m_*, of a crystal will be raised. Thus, one expects to find higher values of *T_m_* for samples which are isothermally crystallized for longer periods of time if the thickening process can take place at *T_x_* in an observable interval of time. Information on the temperature and time dependence of the thickening process has been obtained in this study. Details of the molecular motions involved in the thickening process are not dealt with here. This has, however, been discussed by Reneker [14], who proposes that the requisite chain motion is accomplished by the diffusion of point dislocations along the chain.

By warming polyethylene very slowly, other investigators [12, 15, 16] have attempted to form crystals that melt at or very near to $T_{m}^{0}$. Such a procedure utilizes the processes of recrystallization and isothermal thickening to attain thick lamellae, and thus high melting points. However, polyethylene’s extremely slow crystal growth rate at temperatures above about 133 °C (where the growth nucleus is large) limits the effectiveness of recrystallization in producing high-melting material, and, as will be seen in section 5.1, isothermal thickening tends to become increasingly ineffective with longer annealing times because of a logarithmic time dependence.

### 2.3. Rate of Heat Transfer

**Figure 1:**
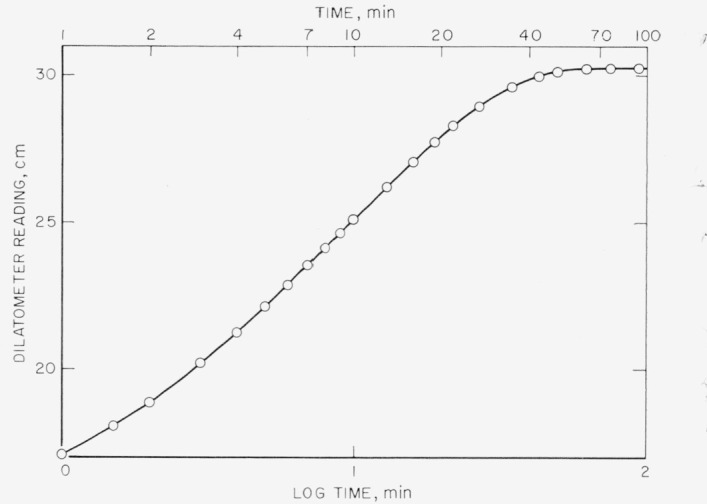
Rate of melting of a ca. 3g polyethylene sample crystallized in mercury dilatometer in 125.0 °C bath for 125 min and then transferred to a 135.20 °C bath.

Another factor that must be taken into consideration in devising the melting procedure is the heat transfer rate in the particular specimens at hand. With the nearly spherical 3 g samples used in this study, more than one hour is needed to achieve a static volume when a highly crystallized sample is transferred from *T_x_* to a temperature at which almost all of its crystalline regions will melt. Figure 1 shows the rate of melting of a sample crystallized in a 125.0 °C bath for 125 min when it is transferred into a bath at 135.20 °C. Temperature measurements with a thermocouple in the sample have shown that thermal equilibrium is not achieved until the material attains a constant volume. The principal causes of the long time needed to reach temperature equilibrium in the sample are the high heat of fusion and low thermal conductivity of polyethylene.

If the melting of a specimen is accomplished by heating it through its melting range at a given rate, the temperature inside the sample will lag behind the bath temperature by an amount dependent on the sample size and geometry, the rate of heating, the degree of crystallinity, and the distribution of melting points. When using the melting procedure given in section 3, the rate of heating was the most critical factor for a given sample, the error in *T_m_* being approximately 27 min times the warming rate in degrees per minute for any rate less than 0.040 deg/min.

Possibly heat transfer is not the only reason for the slowness of the melting at a fixed temperature near the maximum melting point. If one allows sufficient time for the achievement of a static volume part way through the melting range and then raises the temperature slightly, one finds that melting continues for a considerably longer interval than should be necessary in order to reach thermal equilibrium. Matsuo [17], Chiang and Flory [12], and others have noted the same effect.

### 2.4. Other Factors

The phenomena mentioned thus far all tend to cause the observed melting temperature to be greater than it should be for the proper characterization of the original crystallization conditions. On the other hand, two factors may be mentioned which could enter during the course of an investigation and cause the melting points to be lower than the correct values. One is the absorption of solvent. This difficulty was avoided in the present case by the use of mercury-filled dilatometers. The other is decomposition of the polymer. Evidently there was enough antioxidant present in the material studied to prevent degradation in spite of repeated heating to 177 °C. No change in the liquid volume occurred with time; nor was there any significant shift of the crystallization isotherm after repeated melting runs. Thus, barring decompositon and diluent absorption, the procedure which gives the lowest *T_m_* for a given crystallized specimen would seem to be the best one to reflect the size of the crystals as formed under the initial growth conditions.

Whether or not the presence of linear polyethylene of low molecular weight affects the distribution of observed melting points is not known. It appears from the work of Chiang and Flory [12] that unfractionated Marlex 50 does have a larger proportion of low melting crystalline regions than does a fractionated specimen. However, it is possible that much of this difference results from the different times and temperatures required to crystallize the various samples to a high degree.

## 3. Experimental Detail

The material used in this investigation was an unfractionated linear polyethylene (Marlex 50), obtained in the form of small pellets from the Phillips Chemical Company of Bartlesville, Okla. It was used as received except for washing with water and acetone and drying.

Crystallization and melting were carried out in a typical mercury-filled dilatometer. About 3 g of material were used in a 5 ml spherical bulb. The dilatometer capillary was nominally 2 nun in diameter and the height of the meniscus was estimated to the nearest 0.1 mm.

Three Silicone oil bath thermostats were utilized for any given experiment. One bath was for the initial melting of the sample. A temperature of 177 °C for a period of 10 min was found to be sufficient to remove all measurable crystallinity from the sample. No change in the crystallization isotherm was observed as a result of varying the initial melting temperature between 150 and 200 °C. (The crystallization is almost entirely of heterogeneous or pseudohomogeneous origin.) After melting the sample, the dilatometer was transferred to a crystallization bath maintained at *T_x_* ± 0.02 °C by a mercury thermoregulator. Time was measured after the sample had been in the *T_x_* bath for 5 min. After enough time had elapsed to produce the desired crystallinity, the sample was transferred directly to the melting bath which was controlled to a set temperature ± 0.01 °C by a thermistor connected to a bridge circuit. Generally, the melting experiment was begun at a starting temperature just slightly (0.1 to 0.6 °C) below the anticipated melting point in order to avoid recrystallization. A period of 1 hr was allowed at the starting temperature so that nearly all of the crystals which were unstable at that temperature would be melted. Thereafter, the temperature was raised in increments of 0.18° each 30 min until the sample was completely melted. Variations from this scheme were occasionally employed in order to study particular effects.

The temperatures of the baths were measured by a calibrated platinum resistance thermometer. Mercury-in-glass thermometers were found to be unsatisfactory for measuring the bath temperatures because of condensation of the mercury in portions of the capillary above the bath liquid level.

The range of crystallization temperatures employed was 125.0 to 130.0 °C except for a few isolated experiments. The extremely long time required to achieve high crystallinity limited the number of experiments above 130 °C. By the use of a differential thermocouple in one of the dilatometers, it was found that self-heating of the *circa* 3 g specimen during crystallization prevented extension of this range to lower temperatures. Table 1 gives the magnitude of the self-heating near the center of the dilatometer at various times during the crystallization.

The degree of crystallinity, χ, was obtained from the specific volume of the sample, $\bar{V}$, by using the relation $\chi =({\bar{V}}_{l}-\bar{V})/({\bar{V}}_{l}-{\bar{V}}_{c})$ where ${\bar{V}}_{l}=1.1484+9.33\times 10^{-4}T({}^{\circ}C)$ is the measured liquid specific volume and ${\bar{V}}_{c}=0.9940+3.18\times 10^{-4}T({}^{\circ}C)$ is an approximation to Swan’s crystal specific volume as obtained from X-ray studies [18].

## 4. Results of the Melting Studies

The melting temperature customarily reported for a polymeric sample is the highest observed melting point in the distribution of melting points—the last detectable melting point. Obviously, the temperature obtained by this method will vary with the sensitivity of the detector used to observe the presence of crystallinity. When one is interested in determining the effect of crystallization time on the distribution of melting points, the last detectable melting point should not be used; since, for a given distribution in a sample of very low χ, the last detectable melting point will be much nearer to the median point than it will be in a sample of high χ. In order to prevent the fixed and finite sensitivity of the measuring instrument from biasing the melting points for samples of different χ, it has been found to be convenient to normalize the distribution of melting points. This may be accomplished by comparing melting points where the same fraction, *p*, of originally crystallized material remains unmelted. When melting points are being obtained from plots of sample volume against temperature, the most accurate *T_m_* (*p*) correspond to *p*≅0.01. Very low values of *p* do not give precise melting points because the sample volume asymptotically approaches the liquid volume. Large *p* melting points have to be avoided, especially for samples crystallized at low *T_x_*, since recrystallization may occur and significantly alter the fraction melted. In addition, when larger amounts of crystal are undergoing fusion, the temperature of the material is nonuniform, and it is difficult to determine the exact temperature of the crystallites melting at a given instant.

**Figure 2:**
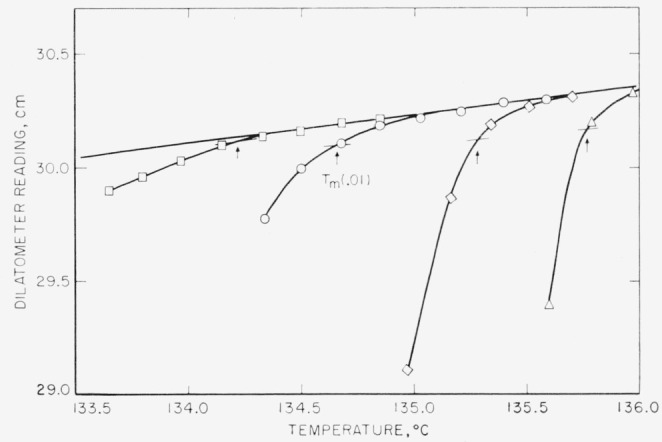
Typical melting curves for samples crystallized in a bath at 126.25 °C. The crystallization times are –□–33 min, –○–70 min, –◊–305 min, and –Δ–9,960 min. The arrows locate the melting temperature where one percent of the original crystallinity remains (*p* = .01).

**Figure 3:**
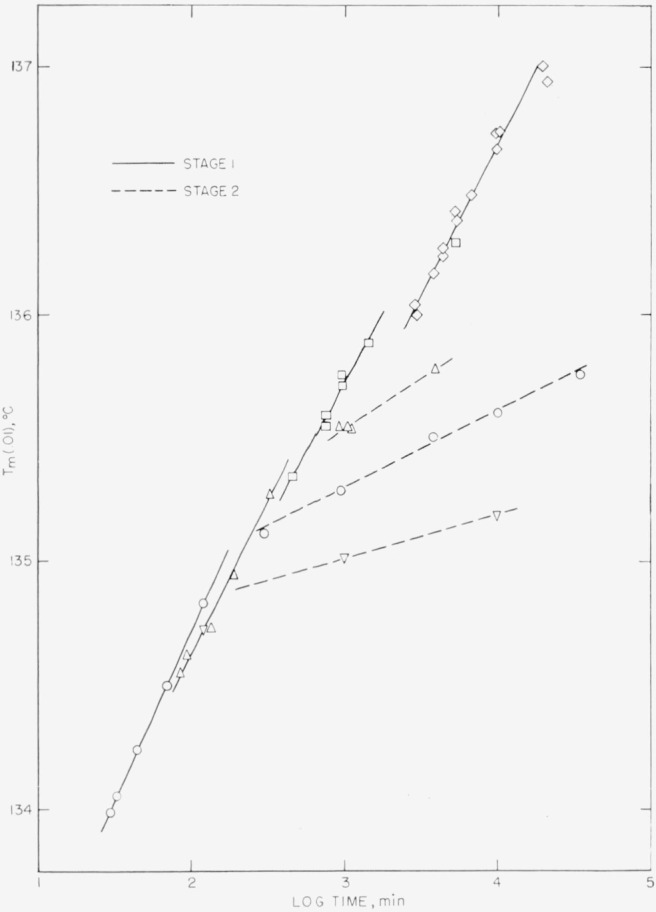
The observed melting temperature, where one percent of the original crystallinity remains, plotted against the logarithm of the crystallization time for various crystallization bath temperatures: –∇–125.0° –○–126.2° –Δ–127.5° –□–128.8°, and –◊–130.0 °C.

Typical melting runs for obtaining *T_m_* (*p*), for *p*=0.01, using one dilatometer at one crystallization temperature, are shown in figure 2. As may be seen, the accuracy of selecting the melting point when 1 percent of the originally formed crystals remain unmelted drops somewhat at very low χ (see left-hand curve in fig. 2). The *T_m_* (0.01) values, corrected for melting rate by the relation given in section 2.3, are reported in table 2 and are plotted in figure 3 as a function of the logarithm of the crystallization time in minutes. These *T_m_* (0.01) melting points clearly show that the high-temperature side of the distribution of melting points is displaced to higher temperatures as the crystallization time is increased.

**Figure 4:**
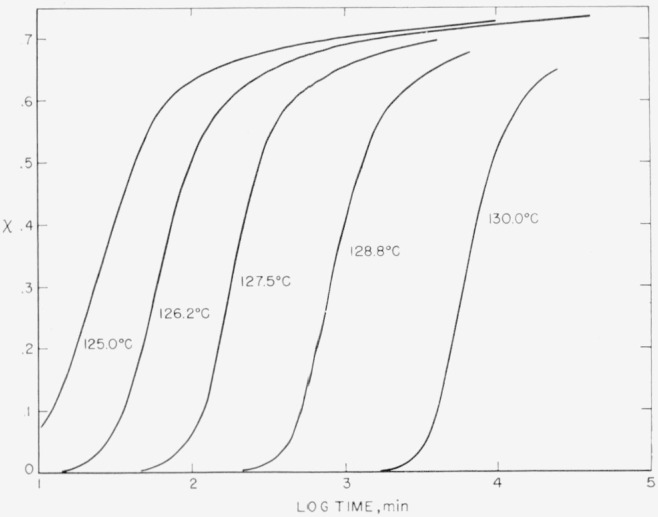
Crystallization isotherms for samples crystallized in baths at the various temperatures shown on the plot. The following induction times, *t*_i_, for the beginning of crystallization were observed: at 125.0 °C, none; at 12C.2 °C, 7 min; at 127.5 °C, 15 min; at 128.8 °C, 90 min; and at 130.0 °C, 450 min.

A comparison of the time scale in figure 3 with that in figure 4, where the crystallization isotherms are plotted, reveals that the change of slope in *T_m_* (0.01) versus log *t* (stage 1→stage 2) occurs at roughly the same time that the isotherm reaches χ*_w_*, the beginning of stage II or secondary crystallization. However, it is not certain that the onset of stage II in a crystallization isotherm, which is a result of massive impingement of spherulites [9], is actually closely connected with the onset of stage 2 in the *T_m_* versus log *t* plot. The apparent increase of the slope for samples crystallized into stage II (dashed lines in fig. 3) with an increase of *T_x_* was confirmed by crystallization of a sample at 125.0 °C for 120 min followed by annealing at 120.0 °C for various lengths of time. The rate of increase of *T_m_* (0.01) with log time of crystallization was found to be 0.05 deg/decade at 120.0 °C, which is almost a factor of 4 smaller than the rate measured at 125.0 °C for samples crystallized into stage II.

**Figure 5:**
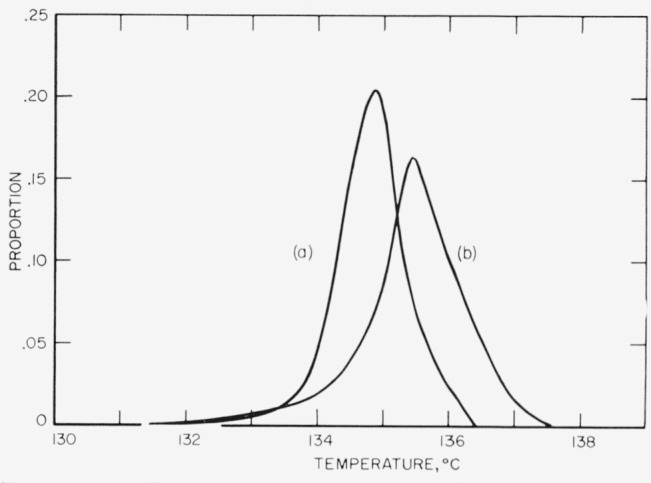
Normalized distributions of the observed melting temperatures of two samples crystallized at 130.0 °C for (a) 3800 min (χ=0.081) and (b) 19,800 mm (χ = 0.635).

In order to obtain the variation of *T_m_*(*p*) with time of crystallization for some values of *p* greater than 0.01, the entire melting range of several samples crystallized at 130.0 °C was recorded. Heating of the bath was carried out at an average rate of 0.019 deg/min. This high crystallization temperature minimizes interference from recrystallization and permits the melting to take place in a short period of time relative to the time at *T_x_.* Two of the normalized distributions of observed melting points are shown in figure 5, where corrections have been made for the heating rate and the amount of material melted.

## 5. Interpretation and Discussion of *T_m_*

### 5.1. Estimation of Lamellar Thickness

The depression from $T_{m}^{0}$ of the melting point of a highly crystalline linear polymer of high molecular weight has been attributed generally to imperfections in the crystals. These may be interior defects, or high energy surfaces, or both. On the assumption that the predominant cause of the depression is the high surface-to-volume ratio, which is known to exist as a result of the lamellar habit, one may estimate the step height of mature crystals which melt at *T_m_.* The relation between the melting point depression, $T_{m}^{0}-T_{m}$, and the thickness, *l*, is given by the thermodynamic formula [6, 8, 19]

$$l=\frac{2\sigma_{e}T_{m}^{0}}{\Delta h_{f}(T_{m}^{0}-T_{m})}$$ (1)

where *σ_e_* is the free energy of formation of the lamellar surface and Δ*h_f_* is the bulk heat of fusion. In deriving this relation, the assumptions are made that (a) the other two dimensions of the crystal are large compared to *l* and (b) the heat capacities of liquid and solid polyethylene are approximately equal in the region of observed melting points.

In order to apply eq (1) to calculate *l* from *T_m_*, it is necessary to estimate $T_{m}^{0}$ and *σ_e_*/Δ*h_f_.* Values of $T_{m}^{0}$ of 141 and 143 °C have recently been obtained by other investigators [20, 21, 22] who extrapolated the melting points of the normal paraffins to infinite chain length. A value of $T_{m}^{0}$ equal to 142 °C is used in the following analysis. Eby and Brown’s measurements [21, 22] of the low angle X-ray spacings and corresponding melting points of several Marlex 50 specimens provide an estimate of *σ_e_*/Δ*h_f_.* This quantity comes from the slope of a plot of *T_m_* against 1/*l* according to a rearrangement of eq (1). A value of *σ_e_*/Δ*h_f_* equal to 2.04×10^−8^ cm was obtained from the results of their measurements, using the smaller of the two low angle spacings as a measure of the lamellar thickness. Geil has shown that this smaller spacing corresponds to the actual lamellar thickness as measured by electron microscopy [5, 23]. (Using Quinn and Mandelkern’s value of Δ*h_f_* [16], as revised in reference [12], we have Δ*h_f_*=2.80×10^9^ erg/cm^3^, which gives *σ_e_*=57 erg/cm^2^ from the previous ratio.) The work of Eby and Brown on Marlex 50, which directly relates *T_m_* and *l*, thus quite directly permits melting point data to be translated into values of the lamellar thickness.

**Figure 6:**
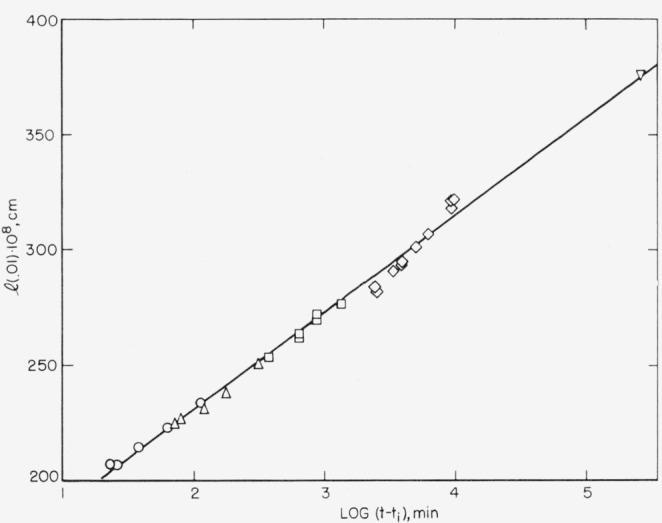
Estimated lamellar thickness as a function of the logarithm of the time of crystallization of the samples (stage 1 data). Melting points shown in figure 3 were used with eq (1) to obtain the estimates of *l*. The crystallization bath temperatures were –○–126.2°, –Δ–127.5°, –□–128.8°, –◊–130.0°, and –▽–131.5 to 133.0 °C.

Estimates of *l*(*p*) for *p*=.01 have been obtained from the *T_m_* (.01) listed in table 2. Figure 6 shows a plot of the *l*(.01) as a function of the time of crystallization. Only values from stage 1 on a *T_m_* versus log *t* plot are given in the figure. (One additional point is included from an eight-month crystallization, during which *T_x_* increased from 131.5 to 133.0 °C. At the time of melting, χ had reached about 0.30.) Here the induction time, *t_i_*, for the appearance of visible crystallinity has been subtracted from the crystallization time as measured from the time the sample reaches its liquid volume at *T_x_.* For specimens with a crystallization time of less than 200 min, it was necessary to make a small correction for the change of lamellar thickness which occurred subsequent to the crystallization time and prior to the time of melting at *T_m_* (0.01). This correction will be considered in section 5.3.

The *l*(*p*) values for *p*=0.01 were obtained by using a value for *σ_e_*/Δ*h_f_* which was calculated from melting point and low angle X-ray measurements on mature polyethylene crystals. If the value of *σ_e_* (equal to 49 erg/cm^2^ [9]) derived from kinetic studies on the nucleation and growth of polyethylene spherulites had been used to obtain *l*, then the estimated *l* values would have been 14 percent smaller.

As figure 6 illustrates, the values of *l* vary linearly, to a good approximation, with the logarithm of the time of crystallization. Hirai et al. [24] and Fisher and Schmidt [25] have found by X-ray diffraction that the long period of polyethylene single crystals, which were grown from solution, increases in a similar manner during annealing. The latter authors also found a similar logarithmic time dependence for the thickening of lamellae in quenched bulk polyethylene which had been annealed at various temperatures. These observations imply that there was initially a rather rapid increase in the thickness of both the melt-grown and solution-grown crystals that occurred prior to the experimentally measured time.

### 5.2. Model for the Thickening Process

Hirai et al. [24] have proposed a nucleation-type model for the thickening of lamellae that leads to an approximately linear increase in *l* with log *t.* They make the observation that a nucleation barrier must be surmounted in order for lamellar thickening to lower the free energy of a crystal of given fixed volume. Below we summarize this approach in slightly revised form. Taking the dimensions of a surface nucleus to be *x* on each side and *g* in height, one has for the change in free energy of a crystal of thickness *l*,

$$\Delta F=4xg\sigma -2x^{2}g\sigma_{e}/l$$ (2)

because of the local increase in thickness at the nucleation site and corresponding local decrease in basal area. Here *σ* is the free energy of formation of the lateral surface per unit area. The location of the minimum in the free energy surface, ΔF*, is independent of *g* but its value is proportional to *g*; thus, the increase in *l* should be accomplished by steps of only 2.54 Å at a time.^2^ After a critical value of *x* has been exceeded, the increase in thickness is as sumed to be propagated rapidly over a rather large area. Just how large an area might depend on external impingements, strain terms, or other complex factors. By differentiation of Δ*F* with respect to *x*, one finds

$$\Delta F*=(2g\sigma^{2}/\sigma_{e})l=Cl$$ (3)

Thus, as an elementary approximation, one has

$$\frac{dl}{dt}=Ae^{-Cl/kT}.$$ (4)

(Hirai et al. calculate that *dl*/*dt*=*A′* exp (*−Cl*/*2kT*), but, by a more rigorous derivation based on the method of Turnbull and Fisher [26], Lauritzen [27] has shown that the correct exponent is given by eq (4).)

At this point we mention that an equation identical in form to eq (4) has been obtained by Peterlin [28] on the assumption that there exists an energy barrier of height *nE* for the lengthwise translation of a chain of *n* carbon atoms to an adjacent crystal lattice site. The rate at which this motion could occur is *dn*/*dt*=*A*″ exp(*−nE*/*kT*). Since *l*=1.27×10^−8^*n*(cm), this equation is similar in form to eq (4). Hirai assumes that the barrier to lengthwise translation is independent of the crystal thickness. (This point will be re-examined later.)

Integration of eq (4) gives

$$l=B\;\log\frac{AC}{kT}+B\;\log(t-t_{0}+\tau_{0}),$$ (5)

where *B*=2.3 *kT*/*C* and $\tau_{0}=(kT/AC)\;\exp(l_{g}^{*}C/kT)$. It is supposed that a crystal born at time *t*_0_ initially has a thickness equal to $l_{g}^{*}$. When *t–t*_0_ is large compared to *τ*_0_—which is the case in the experimentally accessible time range—then

$$l≅B\;\log\frac{2.3A}{B}+B\;\log(t-t_{0}),$$ (6)

or alternatively

$$l≅l_{g}^{*}+B\;\log\frac{t-t_{0}}{\tau_{0}}.$$ (6a)

Equation (6) put in terms of the crystallization time, rather than the existence time, of the crystal by noting that the age of any fraction, *p*, of the crystals in a given sample crystallized to *x_f_* at time *t_f_* (equal to *t*−*t_i_*) is greater than or equal to *t_f_−t*_0_, where *t*_0_ is the time at which χ=χ_0_=*p*χ*_f_.* If there is a monotonie dependence of melting temperature on age as a consequence of isothermal thickening, then the crystals that melt at temperatures higher than *T_m_*(*p*) will have formed earlier than time *t*_0_. Thus, *t_f_−t*_0_ is the estimated age of a crystal melting at *T_m_*(*p*). To a good approximation the isotherms in figure 4 are given by the equation [29]

$$\chi =\chi_{w}[1-e^{-Z(t-t_{i}3/\chi }w],$$ (7)

where *Z* is a constant for a given isotherm. This corresponds to the growth of heterogeneously nucleated spheres. From this equation

$$t_{0}=q^{1/3}t_{f},$$ (8)

where *q*=log (1−χ_0_/χ*_w_*/log (1−*x_f_*/*x_w_*).Substitution for *t*_0_ in eq (6) gives

$$l≅B\;\log\frac{2.3A}{B}+B\;\log\;(1-q^{1/3})+B\;\log\;t_{f}.$$ (9)

since *t_f_* is the crystallization time. A slightly poorer but more convenient approximation results if the “free growth rate,” χ = *Z*(*t−t_i_*)^3^, is assumed in place of eq (7). In this case,

$$l≅B\;\log\frac{2.3A}{B}+B\;\log(1-p^{1/3})+B\;\log\;t_{f}.$$ (10)

Average values of the parameters *A, B*, and *G* may be calculated from the plot in figure 6 by use of eq (10). The slope gives *B*=43.3×10^−8^ cm; *A* is found to be 7.8×10^−6^ cm/sec; and *C* is 2.94×10^−7^ erg/cm for *T*=400 °K. Taking *g* as equal to 2.54×10^−8^ cm, one may obtain *σ*^2^/*σ_e_* = 5.8 erg/cm^2^ from the magnitude of *C.* This value of 5.8 for *σ*^2^/*σ_e_* may be compared to the value of 3.0 that has been obtained from parameters for the nucleation and growth of polyethylene crystals [9]. Various assumptions can be advanced to account for the difference in *σ*^2^/*σ_e_* values, for example: (a) other barriers to the formation of the nucleus, such as that proposed by Peterlin for the lengthwise translation (“sliding diffusion”) of the chains, (b) the existence of restraints on the thickening process (see section 5.4), and (c) the fact that the lateral surface of the nucleus, which presumably rises only 2.54 A above the surrounding crystal, is not typical of the lateral surface of either a primary nucleus or a growth nucleus. The extent to which these factors may be operative is not known, but it is likely that the barrier for lengthwise translation of the chains is dependent on *l.* This translation barrier will probably be more temperature dependent than the nucleation barrier; if it were, it could explain the strong temperature dependence of *B* that Fisher and Schmidt [25] observed. They found that the magnitude of *B* increased by a factor of 3 in the range 120 to 130 *°C.* The data in figure 6 also indicate a positive temperature coefficient for *B.* The 130 °C points give a value of *B* = 64×10^−8^ cm (*σ*^2^/*σ_e_*=3.9 erg/cm^2^) and *A* = 6.5×10^−8^ cm/sec when treated by themselves. When both the nucleation and translation barriers are controlling the thickening rate, the quantity *B* is given by 2.92*kTσ_e_*/(10^8^*Eσ_e_* + 2.54 g *σ*^2^). If *E* decreases with increasing temperature as the lattice expands, then the nucleation barrier would be the limiting rate controlling factor at high temperatures while the translation barrier might be the limiting factor at very low temperatures.

### 5.3. Change of During Melting

**Figure 7:**
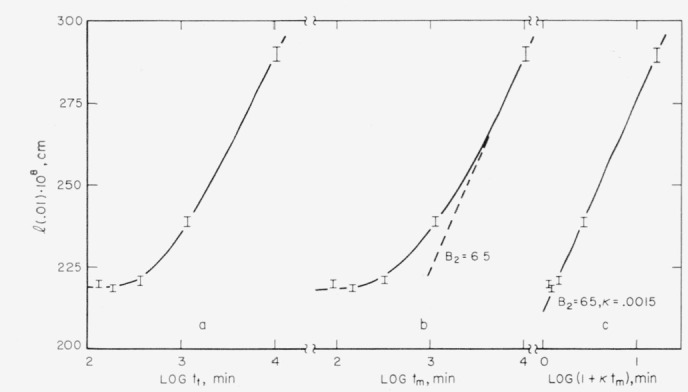
The estimated lamellar thickness, l (.01), for a polyethlene sample crystallized in 126.25 °C hath for 42 min and then melted in time interval *t_m_*: 7a. *Plotted against the logarithm of the total time of crystallization plus melting*, t_t_; 7b. *Plotted against log* t_m_; 7c. *Plotted against log* (*1+κ*t_m_) *where κ is a constant equal to 0.0015.*

Of necessity, samples crystallized at *T_x_*, and then melted using the procedure followed here, contain crystals that have existed for a greater time interval than that measured at *T_x_.* In order to determine the amount of lamellar thickening that occurred during the melting process, a polyethylene sample was crystallized in a 126.25 °C bath for 42 min, then transferred to a bath at 133.64 °C, where it was stored for a time interval *t_s_*, and then melted at a rate of 0.006 deg/min. By repeating the procedure, the data given in table 3 were obtained. Values of *l* (0.01) were derived from the *T_m_* (0.01) by use of eq (1). These values are plotted in figure 7a as a function of the logarithm of the total time of crystallization plus melting, *t_t_.* This plot is not suitable for determining the change in *l* during melting. A probable explanation of the shape of the curve in figure 7a may be found from a consideration of the effect of a change in the parameters *A* and *B* as the temperature is changed from *T_x_* to one near *T_m_* (0.01). Assume that a lamella is crystallized at *T_x_* for a time (*t*_1_*−t*_0_) where the thickening rate is

$$\frac{dl}{dt}=A_{1}e^{-2.3\;l/B_{1}};l_{g}^{*}<l<l_{1},t_{0}<t<t_{1}.$$ (11)

After this time the crystallite is transferred to a higher temperature where it thickens at a rate

$$\frac{dl}{dt}=A_{2}e^{-2.3\;l/B_{2}};\;l>l_{1},t>t_{1}.$$ (12)

When eqs (11) and (12) are integrated and combined one obtains

$$l-l_{1}=B_{2}\log\{1+\kappa (t-t_{1})\}$$ (13)

where $\kappa =(2.3A_{2}/B_{2}){\left[\left(2.3A_{1}/B_{1})(t_{1}-t_{0}+\tau_{0}\right)\right]}^{-B_{1}/B_{2}}$ and

$$\tau_{0}=(B_{1}/2.3A_{1})\exp2.3l_{g}^{*}/B_{1}$$

which is negligible compared to *t*_1_−*t*_0_.

According to eq (13), a plot of *l* versus log (*t−t*_1_) will give a curve whose slope approaches *B*_2_ when *t−t*_1_>>*κ^−1^*. Such a plot is shown in figure 7b where *B*_2_ has been estimated to be about 65×10^−8^ cm. When *B*_2_ is known, *κ* may be estimated from a plot such as that given in figure 7c. The data imply a *κ* of about 0.0015. Once *B*_2_ and *κ* have been determined, eq (13) may be used to obtain the change in *l* (0.01) that occurs during the melting procedure (by successive approximation until *B*_1_ is known). In this way corrections were applied to the *l*(0.01) which were crystallized for less than 200 min, as was mentioned in section 5.1. The maximum correction was 7.5×10^−8^ cm.

All of the quantities in *κ* have been estimated except for *A*_2_. Using *κ*=0.0015, *B*_1_=43×10^−8^ cm, *B*_2_=65×10^−8^ cm, and *A*_1_ = 7.8×10^−6^ cm/sec, one finds that *A*_2_=2.4×10^−5^ cm/sec. This is about the same change in *A* with temperature that Hirai et al. have found for the annealing of polyethylene single crystals [24].

### 5.4. The Distribution of Step Heights and Restraints to Isothermal Thickening

**Figure 8:**
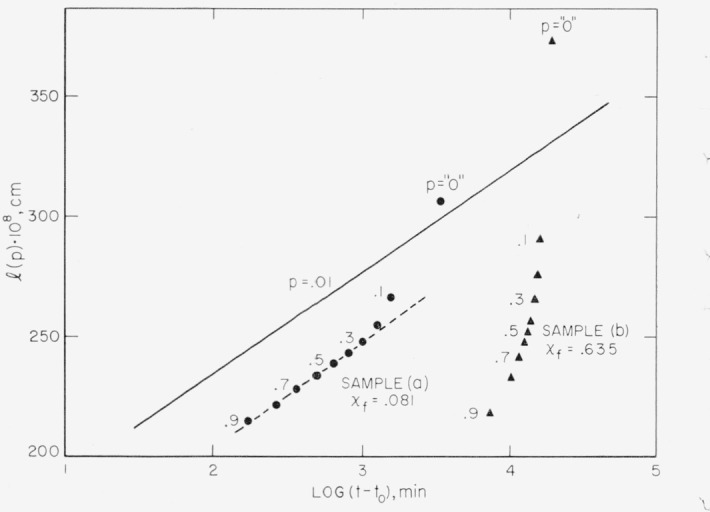
Estimated crystal thickness from *T_m_(p*) for the two distributions shown in figure 5 as a function of the logarithm of the estimated existence time of the crystals. Sample (a) crystallized at 130.0° for 3800 min, ●; sample (b) crystallized at the same temperature for 19,800 min, ▲.

The nucleation theory of lamellar thickening successfully accounts for the time dependence of the values of *l* estimated from the *T_m_* (0.01); however, it does not, in itself, provide a complete explanation of the distributions of melting points shown in figure 5. This may be shown by obtaining the *l*(*p*) for *p*=0, .1, .2, …, 1.0 from the entire melting curves and the corresponding values of *t−t*_0_ from the 130.0 °C crystallization isotherm, assuming a perfect positive correlation between age and melting temperature. These *l* values are plotted in figure 8. (They have been adjusted slightly so that the *l*(0.01) fall exactly on the *l* versus log *t* curve of figure 6.) According to eq (6) all of the *l*(*p*) [points should fall on the same curve. This curve should be essentially a straight line, when *t*−*t*_0_>100 min, having a slope *B* equal to 43.3×10^−8^ cm. It has been drawn as a solid line in figure 8 and is labeled *p*=0.01. Three points of interest are apparent in the plot: (i) about 80 percent of sample (a) has *l*(*p*) which give a slope similar to the *l*(0.01) but which are displaced to lower values (see dashed line); (ii) the *l*(0) points imply a much greater slope than do the *l*(0.01) points; and (iii) the *l*(*p*) for sample (b) fall much below the solid line except for *l*(0). These effects are thought to be the result of two factors which have not been treated in deriving eq (6); namely, the existence of a distribution of step heights around $l_{g}^{*}$ when crystals are first formed from the melt and the existence of restraints to normal lamellar thickening.

Lauritzen and Hoffman [6, 27] have considered the distribution of step heights of growing chain-folded crystals and have found that the probability distribution may be represented by

$$F(l)≅\gamma^{2}(l-l_{gm})e^{-\gamma (l-l_{gm})}$$ (14)

to an approximation sufficient for the present purpose. Here *l_gm_* = 2*σ_e_*/Δ*f* where Δ*f* is the bulk free energy of fusion, *γ* = *2b*_0_*σ*/*kT* (which is taken as a constant), and *b*_0_ is the width of a chain. By assuming that crystals are generated according to χ=*Z*(*t−t_i_*)^3^ with a distribution of step heights given by eq (14), Lauritzen [27] has found the following relation for crystals that have thickened according to eq (4) in a sample crystallized for time *t_f_*=*t−t_i_* when *t*>>*τ*_0_:

$$\begin{array}{l} l^{\prime }=\frac{2.3}{\gamma }\log\frac{3}{p}+\frac{2.3}{\gamma }\log\frac{2\lambda^{3}\gamma \left[l^{\prime }-l_{gm}-B\;\log\left(1+\frac{t_{f}}{\tau_{0}}\right)\right]}{\left(\gamma +\lambda )(\gamma +2\lambda )(\gamma +3\lambda \right)}\\ +\lambda^{3}\frac{8\gamma^{3}+36\gamma^{2}\lambda +44\gamma \lambda^{2}+12\lambda^{3}}{{\left(\gamma +\lambda \right)}^{2}{\left(\gamma +2\lambda \right)}^{2}{\left(\gamma +3\lambda \right)}^{2}}\\ +l_{gm}-B\;\log\tau_{0}+B\;\log t_{f}. \end{array}$$ (15)

Here *λ* equals 2.3/*B*. This equation holds for *p*<~0.02 and may be compared at low *p* with a modification of eq (10), namely,

$$l=B\;\log(1-p^{1/3})+\frac{2}{\gamma }+l_{gm}-B\;\log\tau_{0}+B\;\log t_{f}.$$ (16)

The more detailed treatment by Lauritzen shows that *l*(0.01) should be essentially a linear function of log (*t−t_i_*) as has been observed in figure 6. However, eq (15) shows a stronger dependence of *l* on *p* than does eq (16) for low values of *p.* In order to demonstrate a qualitative agreement between the *l*(*p*) values for low *p* shown in figure 8 and those values predicted by eq (15), we note that the dilatometers used in this study have a fixed limit of sensitivity of about 0.25 mg of polyethylene crystal. This is equivalent to a degree of crystallinity of about 0.00008 and means that the so-called *“p* = 0*”* values in figure 8 actually are *p* = 0.00008/0.081 = 0.001 and *p* = 0.00008/0.635=0.00013 for samples (a) and (b), respectively. Now one may compare the change in the *l*(*p*) predicted by eqs (15) and (16) with that observed experimentally. This is done in table 4 by computing *l*(*p*)*−l*(.02) for several values of *p.* It is seen that the variation of *l* with *p* shown by sample (a) agrees well with that predicted by eq (15). From the foregoing consideration of the effect of a distribution of step heights on *l*(*p*) in a crystalline specimen, one may suppose that in a low χ sample the *T_m_* (*p*) for large *p* generally reflect the melting of crystals initially formed near the average of the step-height distribution. At lower values of *p*, fewer and fewer crystals remain in the sample so that the observed melting points correspond to the larger crystals in the original distribution. The strong dependence of *l* on *p* for low values of *p* thus explains the strong upswing in *l*(*p*) for *p*<0.1 that is seen in figure 8 and which is a result of the “tail” on the melting curves (see figure 2). This “tail” on the melting curve near the liquidus is due primarily to the corresponding tail on the high end of the *F*(*l*) distribution, which persists at all ages.

The fact that the *l*(*p*) for high *p* in sample (b) fall appreciably below the *p*=0.01 curve may be interpreted as showing the existence of restraints to normal isothermal thickening of the lamellae. These restraints are probably of varying degree, but they may also vary with χ (the stage of development of the “spherulites”)· They can be visualized to occur as the result of external impingement of adjacent lamellae, which may be most severe in regions of secondary surface nucleation, branching, spiral dislocation, or numerous interlamellar tie molecules; and/or they may result from internal build-up of strain associated with the large mass transport required for lamellar thickening at constant volume. Irrespective of the cause, the existence of restraints to normal thickening would destroy the prefect correlation between age and melting point which was assumed in obtaining the coordinates for the points in figure 8. Because of the possibility of residual restraints on the thickening process, the true value of *B*—estimated previously from the slope of *l*(0.01) versus log *t*—may be greater than that shown in figure 6, but the similarity of this slope with that defined by high *p*-values of *l* for sample (a) suggests that *B* is not appreciably biased by restraints.

From the foregoing, it is reasonable to suppose that the transition from stage 1 to stage 2 on a *T_m_* or *l* versus log *t* plot (compare figs. 3 and 6) is a result of retardation to thickening in the *c* crystallographic direction of the older lamellae in the spherulites. Conversely, the transition from stage I to stage II in a crystallization isotherm is thought to result from retardation to growth in the *a* and *b* directions, e.g., impingements of lamellae at spherulite boundaries.

### 5.5. Estimates of $l_{g}^{*}$ from *T_m_*(1.0)

Of particular interest is the fact that the smallest estimated values of *l*, which were obtained from the complete melting curves of samples crystallized at 130.0 °C, are close to the value of $l_{g}^{*}$ predicted by theories for polymeric crystal growth with chain folding [6, 7] if the “thermodynamic” value of the surface free energy is employed (*σ_e_*=57 erg/cm^2^). Similar observations have been made for other crystallization temperatures as are shown in table 5. The discrepancy that appears at the highest crystallization temperature could imply that some thickening of the *l*(1.0) has occurred during the very long storage at *T*_x_.

The crystals melting at *T_m_*(1.0) are assumed to be the thinnest (and therefore lowest melting) of all those in the specimen because of a short time of existence and/or a very highly impeded thickening. It is conceivable that they could be low melting because of a defective internal structure as a result of the incorporation of short branches into the crystal. But Keith [30] has found that branched structures tend to be rejected at the growing boundary. The crystals in samples that have been crystallized slowly to a low χ should be especially free from internal defects.

There is a strong implication from the logarithmic time dependence of the lamellar thickness, together with the values found for *l*(1.0), that polyethylene crystals are initially formed in a growth process having a thickness near that of the theoretical critical-size growth nucleus, $l_{g}^{*}$. This thickness evidently increases very rapidly at first, at a rate inversely proportional to (*t*−*t*_0_+*τ*_0_), and eventually gives a thickness of approximately $2l_{g}^{*}$ in the region that is experimentally accessible for study by low- angle X-ray diffraction or by electron microscopy of surface replicas.

### 5.6. Extrapolation of *T_m_* Versus *T_x_* To Obtain

The experimentally measured increase in *T_m_* with increasing crystallization time, when interpreted as in increase of lamellar thickness, helps to elucidate the extrapolation procedure proposed by Hoffman and Weeks [8] for obtaining $T_{m}^{0}$. In the application of this method one assumes that the thicknesses of the larger mature lamellae (in samples crystallized to a given value of χ and then melted without recrystallization) are dependent only on the crystallization temperature and may be closely approximated by $\beta l_{p}^{*}$, where *β* is a constant and $l_{p}^{*}$ is equal to 4*a_e_*/Δ*f.* As an example, if one obtains the crystallization times of samples crystalized to a χ of 0.10 from the isotherms in figure 4 and determines the corresponding values of *l* from figure 6, then one finds that *β* increases from 0.98 to 1.04 in the temperature range 126.5 to 130.0 °C. A similar change in *β* occurs using *l*(0) instead of *l*(0.01) values. The increasing value of *β* causes an appreciable error in the estimate of $T_{m}^{0}=145.5\;{}^{\circ}C$ that is obtained by linear extrapolation of *T_m_*(0) versus *T_x_* to the intersection with the line *T_m_*(0)= *T_x_.*

It appears that, for polyethylene, a better extrapolation for obtaining $T_{m}^{0}$ than the one suggested by Hoffman and Weeks is one based on eq (1), where the observed melting point is plotted as a function of the reciprocal of the lamellar thickness. When Eby and Brown’s data [21, 22] are plotted in this manner, one obtains a value of of about 143.5 °C by extrapolation of either of the two long spacings which they report. If *p*=0.50 melting points are used instead of the last detectable melting points, then the estimate of is about 1.5 degrees lower. The *T_m_* (0.50) probably correspond more closely to the *l’*s obtained from low-angle X-ray diffraction than do the last detectable melting points. This is consistent with the value $T_{m}^{0}=142\;{}^{\circ}C$ used in the preceding sections and corresponds well with the value obtained from extrapolation of paraffin data.

### 5.7. Summary of the Analysis

Eby and Brown’s [21, 22] experimental verification of eq (1) has shown that the principal cause of a sample’s melting below $T_{m}^{0}$ is the thinness of its lamellar crystals. Their measurements provide a value for *σ_e_*/Δ*h_f_*, but other estimates of *σ_e_* [9] and Δ*h_f_* [16] could have been used just as well for estimating lamellar thickness from the melting point. A study of the melting temperature of polyethylene specimens has shown that isothermal thickening occurs and that it has a logarithmic time dependence. On the basis of a nucleation model of the thickening process, about half of the observed rate of change of *l* is accounted for between 125 and 130 °C. Another contributing factor is presumed to result from a barrier (to the lengthwise translation of the chains) that depends on chain length [28]. There is evidence to show that this barrier decreases with increasing temperature, allowing substantially more than one half of the rate of change of *l* to be explained by the nucleation model near and above 133 °C. When the theory is extended by the inclusion of a distribution of step heights around $l_{g}^{*}$, one can quantitatively attribute the entire distribution of melting points, observed for a sample of low χ, to differences in the ages of the crystallites. However, as the degree of crystallinity increases, it is evident that something restrains a fraction of the crystals from thickening at their normal rate. This impeded fraction increases with the time of crystallization until, for the samples crystallized into stage II, even the last one percent of the crystals exhibit a diminished thickening rate. Values of $l_{g}^{*}$ close to those predicted by recent theories of growth with chain folding are obtained from *T_m_* (1.0) data. An estimate of $T_{m}^{0}$ (equal to 142 °C) has been obtained from Eby and Brown’s data by the use of eq (1).

## Figures and Tables

**Table 1:** Self-heating during crystallization of circa 3 g polyethylene specimens in mercury dilatometer

Bath temperature	Differential temperature	Time of crystallization	Degree of crystallinity
			
°*C*	°*C*	*min*	
125.00	0.69	12	0.10
	a 1.05	23	.29
	0.13	100	.63
126.25	.59	35	.10
	a.77	58	.29
	.54	99	.50
127.50	.26	119	.10
	a.33	168	.27
	.21	278	.50
128.75	.06	520	.10
	a.09	730	.26

a Maximum differential temperature during the crystallization.

**Table 2:** The observed melting temperature of polyethylene when 1 percent of the original crystallinity remains

Crystallization temperature	Time of crystallization	Warming rate	*T_m_* (0.01)
			
*°C*	*min*	*deg/min*	*°c*
125.00–126.05	120	0.006	134.73
	1,204	.006	135.02
	10,000	.012	135.19
126.25–127.02	30	.003	133.99
	33	.006	134.06
	45	.006	134.24
	70	.006	134.50
	120	.006	134.83
	305	.006	135.12
	950	.006	135.29
	3,820	.006	135.51
	9,960	.006	135.61
	34,200	.006	135.76
127.50–127.83	87	.006	134.55
	95	.006	134.62
	135	.006	134.73
	191	.006	134.94
	330	.006	135.27
	920	.006	135.55
	1,040	.000	135.54
	1,040	.000	135.54
	3,890	.006	135.78
128.75–128.84	465	.004	135.35
	745	.006	135.55
	745	.006	135.59
	970	.006	135.72
	970	.006	135.76
	1,410	.006	135.89
	5,300	.006	136.29
130.00	2,820	.006	136.04
	2,930	.007	136.00
	3,800	.019	136.17
	4,320	.007	136.27
	4,330	.000	136.24
	5,280	.019	136.42
	5,380	.006	136.38
	6,680	.006	136.48
	9,650	.006	136.73
	9,910	.007	136.67
	10,200	.030	136.74
	19,800	.019	137.01
	21,200	.006	136.94

**Table 3:** Melting temperature of polyethylene sample stored at 133.64 °C for various lengths of time^a^

Time of storage, *t_8_*	Total melting time, *t_m_*	Corrected *T_m_*(0.01)^b^	*l*(.01)
			
*min*	*min*	*°C*	*cm*
(not stored)	91	134.30	220.0×10^−8^
23	148	134.25	218.5
148	328	134.34	221.0
903	1143	134.91	239.0
9, 906	10,331	136.16	290.0

a The sample was initially crystallized in a 126.25 °C bath for 42 min.

b *T_m_* (0.01) observed minus 0.16 °C heating rate correction.

**Table 4:** Comparison of *l(p)−l(0.02*) observed with tha predicted by eqs (15) and (16)

*p*	*l*(*p*)*—l*(.02) by eq (15)	*l*(*p*)*—l*(. 02) by eq (16)	Observed *l*(*p*)—*l*(.02)	
			Sample (a)	(b)
				
	*cm*	*cm*	*cm*	*cm*
0.01	4.4×10^−8^	1.4×10^−8^	5.5×10^−8^	10.0×10^−8^
a.0010	19.1	4.0	17.5	………
b.00013	31.5	5.0	………	53.5

a *p*=“O” for sample (a).

b *p*=*“*O*”* for sample (b).

**Table 5:** Comparison of *l(1.0*) with the theoretical value of a growth nucleus of critical size, $l_{g}^{*}$

Crystallization temperature	Time of crystallization	(1.0)	al^*^_g_
			
*°C*	*min*	*cm*	*cm*
125.00–126.05	120	113×10^−3^	111–117×10^−8^
	120	107	(94–99)
	b120	100	
130.00	3800	158	152
	5280	168	(128)
	19,800	160	
131.60–133.00	288,000	218	174–199
			(146–167)

a The values of $l_{g}^{*}$ are calculated as $l_{g}^{*}=\frac{2\sigma_{e}T_{m}^{0}}{\Delta h_{f}(T_{m}^{0}-T_{x})}+\frac{kT_{x}}{b_{0\sigma }}$ after Lauritzen and Hoffman [6], where *k* is Boltzmann’s constant and *b*_0_ is the width of a chain. The values $T_{m}^{0}=415\;{}^{\circ}K$, *σ_e_*=57 erg/cm^2^, *σ*=12 erg/cm^2^ [9], Δ*h_f_*=2.8×10^9^ erg/cm^3^, and *b*_0_=4.1×10^−8^ cm were used. The range of $l_{g}^{*}$ values shown refers to the temperature range in the first column. Similar results are obtained using Price’s formulation of $l_{g}^{*}$ [7], Values in parentheses calculated with *σ_e_*=49 erg/cm^2^ from kinetic data [9].

b Three heating rates w^7^ere used to melt the preceding samples; namely, 0.030, 0.060, and 0.120 deg/min.

## Footnotes

1 Figures in brackets indicate the literature references at the end of this paper.

2 This assumes a lengthwise translation of 5.08 Å by one chain in order to reform the fold with the same configuration that it had previously. A translation of half this amount may be possible but would produce a higher energy fold.
